# Identification of TGF-β signaling-related molecular patterns, construction of a prognostic model, and prediction of immunotherapy response in gastric cancer

**DOI:** 10.3389/fphar.2022.1069204

**Published:** 2022-11-18

**Authors:** Cheng Zeng, Rong He, Yuyang Dai, Xiaohuan Lu, Linghui Deng, Qi Zhu, Yu Liu, Qian Liu, Wenbin Lu, Yue Wang, Jianhua Jin

**Affiliations:** ^1^ Department of Oncology, Wujin Hospital Affiliated with Jiangsu University, Changzhou, Jiangsu, China; ^2^ Department of Oncology, Wujin Clinical College of Xuzhou Medical University, Changzhou, Jiangsu, China; ^3^ Department of Medical Oncology, Shanghai Tenths People’s Hospital, School of Medicine, Tongji University, Shanghai, China; ^4^ School of Medicine, Jiangsu University, Zhenjiang, Jiangsu, China; ^5^ Department of Gastrointestinal Surgery, Union Hospital, Tongji Medical College, Huazhong University of Science and Technology, Wuhan, Hubei, China; ^6^ Department of Oncology, The First Affiliated Hospital of Soochow University, Suzhou, Jiangsu, China; ^7^ Department of Internal Medicine, School of Medicine, Dalian Medical University, Dalian, Liaoning, China; ^8^ Cancer Institute, Xuzhou Medical University, Xuzhou, Jiangsu, China

**Keywords:** gastric cancer, TGF-β, molecular pattern, prognosis, tumor microenvironment, immunotherapy

## Abstract

**Background:** TGF-β signaling pathway plays an essential role in tumor progression and immune responses. However, the link between TGF-β signaling pathway-related genes (TSRGs) and clinical prognosis, tumor microenvironment (TME), and immunotherapy in gastric cancer is unclear.

**Methods:** Transcriptome data and related clinical data of gastric cancer were downloaded from the Cancer Genome Atlas (TCGA) and Gene Expression Omnibus (GEO) databases, and 54 TSRGs were obtained from the Molecular Signatures Database (MSigDB). We systematically analyzed the expression profile characteristics of 54 TSRGs in 804 gastric cancer samples and examined the differences in prognosis, clinicopathological features, and TME among different molecular subtypes. Subsequently, TGF-β-related prognostic models were constructed using univariate and least absolute shrinkage and selection operator (LASSO) Cox regression analysis to quantify the degree of risk in each patient. Patients were divided into two high- and low-risk groups based on the median risk score. Finally, sensitivity to immune checkpoint inhibitors (ICIs) and anti-tumor agents was assessed in patients in high- and low-risk groups.

**Results:** We identified two distinct TGF-β subgroups. Compared to TGF-β cluster B, TGF-β cluster A exhibits an immunosuppressive microenvironment with a shorter overall survival (OS). Then, a novel TGF-β-associated prognostic model, including SRPX2, SGCE, DES, MMP7, and KRT17, was constructed, and the risk score was demonstrated as an independent prognostic factor for gastric cancer patients. Further studies showed that gastric cancer patients in the low-risk group, characterized by higher tumor mutation burden (TMB), the proportion of high microsatellite instability (MSI-H), immunophenoscore (IPS), and lower tumor immune dysfunction and exclusion (TIDE) score, had a better prognosis, and linked to higher response rate to immunotherapy. In addition, the risk score and anti-tumor drug sensitivity were strongly correlated.

**Conclusion:** These findings highlight the importance of TSRGs, deepen the understanding of tumor immune microenvironment, and guide individualized immunotherapy for gastric cancer patients.

## Introduction

Gastric cancer is a highly heterogeneous malignant tumor of the digestive system, ranking fifth in incidence and third in mortality worldwide (Smyth et al., 2020). As the early symptoms of gastric cancer are not obvious, some patients have already entered the middle and late stages with poor prognostic when diagnosed (Wei et al., 2020). In recent years, with the application of targeted drugs such as trastuzumab in clinical treatment, the prognosis of HER-2-positive patients with advanced gastric cancer has improved (Zhu et al., 2021). However, the overall prognosis of gastric cancer is still disappointing (Patel and Cecchini, 2020).

Immune checkpoint inhibitors (ICIs) bring new hope to tumor patients due to their significant efficacy and low side effects. However, the response rate of immunotherapy for patients with advanced gastric cancer is less than 30% (Chen et al., 2022), which limits their use in clinical treatment. Studies have shown that the tumor microenvironment (TME) plays a vital role in tumor development and can influence the response rate of ICIs(Zhang and Zhang, 2020). Several biomarkers reflecting the TME, such as tumor mutation burden (TMB), microsatellite instability (MSI), the density of tumor-infiltrating lymphocytes (TILs), and PD-L1 expression, have been found to correlate with the therapeutic efficacy of ICIs(Rizzo et al., 2021; Niu et al., 2022). Tumor cells with high microsatellite instability (MSI-H) have an increased TMB and generate new antigens due to unrepaired mis-replicated DNA, which allows more TILs to infiltrate and thus respond better to ICIs(Lizardo et al., 2020). In addition, patients with high PD-L1 expression have higher response rates to ICIs and longer survival time in most tumors (Ni et al., 2021). Most biomarkers reflect only one aspect of the TME. Recently, some investigators have used transcriptomic data to systematically assess the TME with the help of bioinformatics approaches to screen for different immune phenotypes and thus predict the response rate to ICIs. For example, Zhang et al. (2020) used transcriptomic data from multiple m6A regulators to identify three m6A modification patterns associated with immune phenotypes and to construct an m6A scoring system to predict immunotherapy response.

TGF-β can be produced by most cells through autocrine and paracrine forms, such as tumor cells, stromal cells, and immune cells (Ungefroren, 2019). TGF-β signaling pathway plays a vital role in embryonic development, tumor progression, and immune response (Morikawa et al., 2016; Kim et al., 2021). In early tumor cells, the TGF-β signaling pathway can inhibit proliferation, induce cell cycle arrest and apoptosis, and is considered a tumor suppressor (Colak and Ten Dijke, 2017; Garcia-Rendueles et al., 2017). However, in advanced tumor cells, the TGF-β signaling pathway regulates tumor recurrence and metastasis through mechanisms such as promoting angiogenesis, inducing epithelial-mesenchymal transition (EMT), regulating genomic instability, and immune escape (Colak and Ten Dijke, 2017; Garcia-Rendueles et al., 2017). In addition, the collagen fibers induced by activation of the TGF-β signaling pathway in fibroblasts in the TME restrict the infiltration of T cells into tumor cells, which in turn inhibits the body’s anti-cancer immune response and is regarded as an immunosuppressive cytokine (Batlle and Massagué, 2019; Zhao et al., 2020a). Currently, most studies focus on only one or two genes in the TGF-β signaling pathway, while tumor development is often the result of a large number of genes interacting together. Therefore, it is necessary to systematically analyze the relationship between multiple genes in the TGF-β signaling pathway and the TME to discover new and different immune phenotypes and screen people sensitive to immunotherapy for more precise treatment.

In this study, 804 gastric cancer samples were obtained from TCGA and GEO databases, and 54 TSRGs were collected from MSigDB. We analyzed the expression levels and gene mutation characteristics of 54 TSRGs in gastric cancer and classified gastric cancer patients into two distinct TGF-β subgroups based on the expression levels of the 54 TSRGs. Subsequently, three gene subgroups were identified based on the differentially expressed genes (DEGs) between the two distinct TGF-β subgroups. Next, we constructed and validated a prognostic model, which can predict the prognosis of gastric cancer patients, paint a picture of immune infiltration, and predict ICIs response rates and antitumor drug sensitivity.

## Materials and methods

### Data collection

Gene expression data, somatic mutation data, copy number variation (CNV) data, and corresponding clinicopathological information of gastric cancer patients were downloaded from the TCGA database (https://portal.gdc.cancer.gov/). The GSE84337 dataset was obtained from the GEO database (https://www.ncbi.nlm.nih.gov/geo/). After excluding patients with missing survival time, 804 samples were included in this study, 371 from the TCGA-STAD dataset and 433 from the GSE84437 dataset. To eliminate batch effects of different datasets, we converted fragments per kilobase million (FPKM) values of the TCGA-STAD dataset to transcripts per kilobase million (TPM) and merged two datasets using the ComBat algorithm of the R package sva (Leek et al., 2012). 54 TSRGs were obtained from the MSigDB (HALLMARK_TGF_BETA_SIGNALING) (Supplementary Table S1) (Yu et al., 2022).

### Differential expression and mutational analysis of TSRGs

We performed differential expression analysis of 54 TSRGs in gastric cancer samples and normal samples using R package limma with the adjusted *p* < 0.05 and | log2 FC)|>1 (Ritchie et al., 2015). The protein-protein interaction network of 54 TSRGs was constructed in the STRING database (https://string-db.org/). R package maftools was utilized to map the somatic mutation waterfall of 54 TSRGs in gastric cancer patients (Mayakonda et al., 2018). Lastly, we calculated the CNV gain or loss percentage of 54 TSRGs in gastric cancer patients and analyzed the chromosomal location using the R package RCircos (Zhang et al., 2013).

### Consensus clustering analysis of TSRGs

We first extracted the expression of 54 TSRGs in 804 samples and then performed consensus unsupervised clustering analysis based on 54 TSRGs expression levels using the R package ConsensusClusterPlus (Wilkerson and Hayes, 2010). PCA was performed to visualize the distribution between the two different TGF-β subgroups. To explore the clinical significance of different TGF-β subgroups, we performed Kaplan–Meier survival analysis using the R package survival and survminer (Wang et al., 2020). In addition, we mapped the expression heat map of 54 TSRGs using the R package pheatmap in conjunction with the clinicopathological features of the patients.

### TGF-β-based subtype TME analysis

To explore the differences in TME between TGF-β subgroups, we first analyzed the stromal score, immune score, and ESTIMATE score between two subgroups using the ESTIMATE algorithm. We analyzed the differences in the expression of critical immune checkpoints such as PD-1, PD-L1, and CTLA-4 between the two subgroups. Subsequently, we calculated the infiltration level of 22 immune cells in each sample using the CIBERSORT algorithm (Newman et al., 2015) and analyzed the abundance of immune cell infiltrates between the two subgroups using the single sample gene set enrichment analysis (ssGSEA) algorithm (Zeng et al., 2022). In addition, gene set variation analysis (GSVA) was performed with the hallmark gene set (h.all.v7.5.1.symbols) to investigate the differences in TGF-β subgroups in signaling pathways (Hänzelmann et al., 2013).

### Gene consensus clustering analysis of TGF-β pattern-related DEGs

To identify DEGs in the distinct TGF-β subgroups, R package limma was utilized with |log2-fold change (FC)| ≥ 1 and adjusted *p* < 0.05. Based on the DEGs, we performed gene ontology (GO) enrichment analysis and kyoto encyclopedia of genes and genomes (KEGG) signaling pathway analysis. We performed a clustering analysis based on the expression of DEGs and performed a Kaplan–Meier survival analysis among gene subgroups. In addition, we combined TGF-β subgroups, gene subgroups, and clinicopathological features of patients to map the expression heat map of DEGs.

### Construction and validation of the risk model for gastric cancer

To quantify the degree of risk for each patient, we constructed a risk model based on DEGs. First, we performed univariate regression analysis to screen DEGs associated with the prognosis of gastric cancer patients. Second, we randomly divided the patients into training and testing sets in a 1∶1 ratio (Qing et al., 2022). The training set is used to construct the risk model, and the testing set and the entire set are used to validate the risk model. Third, the LASSO Cox regression analysis was executed in the training set to reduce overfitting genes with 10-fold cross-validation and 1000 repeated times (Tibshirani, 1997). Finally, we performed a multivariate regression analysis using the genes screened by the LASSO regression analysis and calculated the risk score for each patient according to expression levels and regression coefficients of genes. The formula was as follows: Risk score = β _gene1_ × exp _gene1_ + β _gene2_ × exp _gene2_ + … + β _genen_ × exp _genen_ (Qing et al., 2022). Patients were divided into high- and low-risk groups based on the median risk score. Furthermore, we analyzed the relationship between the TGF-β cluster, gene cluster, risk score, and survival status using the R package ggalluvial and the differences in risk scores between distinct subgroups (Zeng et al., 2022). In the training and validation sets, we performed Kaplan-Meier survival analysis with the R package survminer and survival (Wang et al., 2020) and ROC curve analysis with the R package timeROC (Zeng et al., 2022), respectively.

### Subgroup analysis based on available clinicopathological characteristics

To explore the performance power of the risk score among different subgroups of clinicopathological characteristics, we first analyzed the correlation between risk scores and clinicopathological characteristics using the Student’s *t*-test. In addition, Kaplan–Meier survival analysis was performed in different subgroups stratified by age (≤65 years or >65 years), sex (female or male), T stage (T1-2 or T3-4), and N stage (N0 or N1-3).

### Independent prognostic and nomogram analysis

Univariate and multivariate Cox regression analyses were performed to explore whether the risk score could be an independent prognostic factor for gastric cancer patients in the training, testing, and entire set, respectively. Age, gender, tumor size (T), lymph node metastasis (N), and risk score were included for analysis. In addition, we constructed a nomogram integrated the risk score and clinicopathological factors to predict the survival of gastric cancer patients at 1-, 3-, and 5-year using R package rms in the training set, testing set, and entire set, respectively (Zeng et al., 2022). Calibration curves were plotted to determine the performance of the nomograms in predicting OS.

### Investigation of the immune landscape

To explore the differences in the tumor immune microenvironment between high- and low-risk groups of gastric cancer patients based on the risk model, we first analyzed the stromal score, immune score, and ESTIMATE score between the two groups using the ESTIMATE algorithm. Then, we analyzed the Spearman correlation between the risk score and immune cells using seven methods, including the XCELL, TIMER, QUANTISEQ, MCPCOUNTER, EPIC, CIBERSORT-ABS, and CIBERSORT algorithms (Zeng et al., 2022). We further analyzed the Spearman correlation between the expression of 5 genes in the model and immune cells. In addition, the ssGSEA was subjected to calculate the infiltrating immune cells’ scores and assess the activity of immune-related pathways between high- and low-risk groups using the R package gsva (Hänzelmann et al., 2013). Finally, we analyzed the expression levels of immune checkpoint-related genes between high- and low-risk groups.

### Immunotherapy response and antitumor drug sensitivity

TMB(Rizzo et al., 2021), MSS(Rizzo et al., 2021), IPS(Wu et al., 2021), and TIDE (Zeng et al., 2022) scores were considered markers to predict immunotherapy response. First, we downloaded the mutation data of gastric cancer patients in MAF format from the TCGA database and annotated them using the R package maftools (Mayakonda et al., 2018), and subsequently analyzed the correlation between the risk score and TMB as well as the mutated genes common to patients in high- and low-risk groups. Second, we downloaded IPS and MSS data from the TCIA database (http://tcia.at/) for gastric cancer patients and analyzed the differences between patients in high- and low-risk groups. Finally, we analyzed the response rate of gastric cancer patients to immunotherapy based on the TIDE website (http://tide.dfci.harvard.edu/).

Next, we used the R package pRRophetic to calculate the half inhibitory centration (IC50) of antitumor drugs for each patient and analyzed the differences in sensitivity to antitumor drugs between patients in high- and low-risk groups (Geeleher et al., 2014).

### Statistical analysis

R software (version 4.1.2) and related R packages were utilized for statistical analyses. The Wilcoxon test was used to compare clinicopathological characteristics, immune status, TMB, IPS, TIDE scores, and IC50 values between different groups. Kaplan-Meier curves were used to compare OS between different groups. Univariate and multivariate Cox regression analyses were used to analyze independent prognostic factors. ROC curves and nomograms were used to evaluate the predictive power of the risk model. *p* < 0.05 was considered statistically significant. *p < 0.05; **p < 0.01; ***p < 0.001.

## Results

### Differential expression and genetic variation landscape of TSRGs in gastric cancer

**FIGURE 1:**
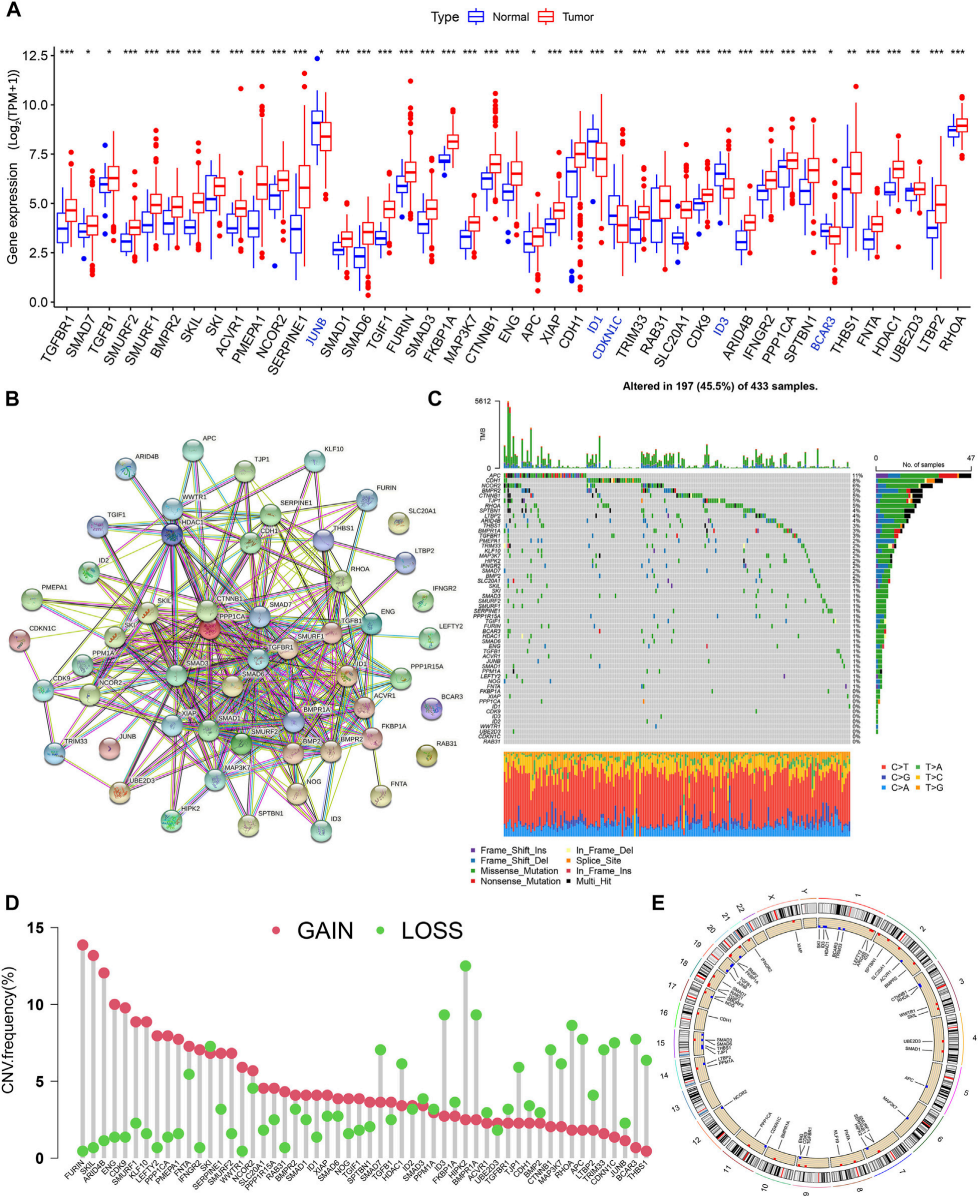
Genetic mutational characteristics of TSRGs in gastric cancer, **(A)** Differential expression analysis of TSRGs in gastric cancer and normal tissues. **(B)** Protein-protein interaction network analysis of TSRGs in the STRING database. **(C)** Mutation frequency analysis of TSRGs in gastric cancer. **(D)** Frequencies of CNV gain, loss, and non-CNV among TSRGs. **(E)** Locations of CNV alterations in TSRGs on chromosomes. TSRGs, TGF-β signaling related genes; CNV, copy number variant; *p < 0.05; **p < 0.01; ***p < 0.001.

The design idea of this study is shown in Supplementary Figure S1. We first performed differential expression analysis of 54 TSRGs in gastric cancer tissues and normal gastric tissues. We obtained 43 DEGs with the adjusted *p* < 0.05 and | log2 FC)|>1, of which JUNB, ID1, CDKN1C, ID3, and BCAR3 were lowly expressed in gastric cancer tissues, and the remaining DEGs were highly expressed in gastric cancer tissues (Figure 1A). Protein-protein interaction network analysis based on the String database revealed a close linkage between most genes (Figure 1B). Next, we explored the somatic mutation levels and the frequency of CNVs alteration in 54 TSRGs in gastric cancer patients. The waterfall plot in Figure 1C shows that 197 (45.5%) of the 433 samples had TSRG mutations. Among them, APC (11%) had the highest mutation frequency, followed by CDH1 (8%) and NCOR2 (6%). Missense mutations are the most common form of mutation in TSRGs. We also investigated the frequency of CNVs alterations of TSRGs and found that FURIN, SKIL, and ARID4B had the most significant copy number increase, while HIPK2, ID3, and BMPR1A had the most significant copy number deletion (Figure 1D). Figure 1E shows the site of CNVs of TSRGs on chromosomes.

### Identification of TGF-β subgroups in gastric cancer

**FIGURE 2:**
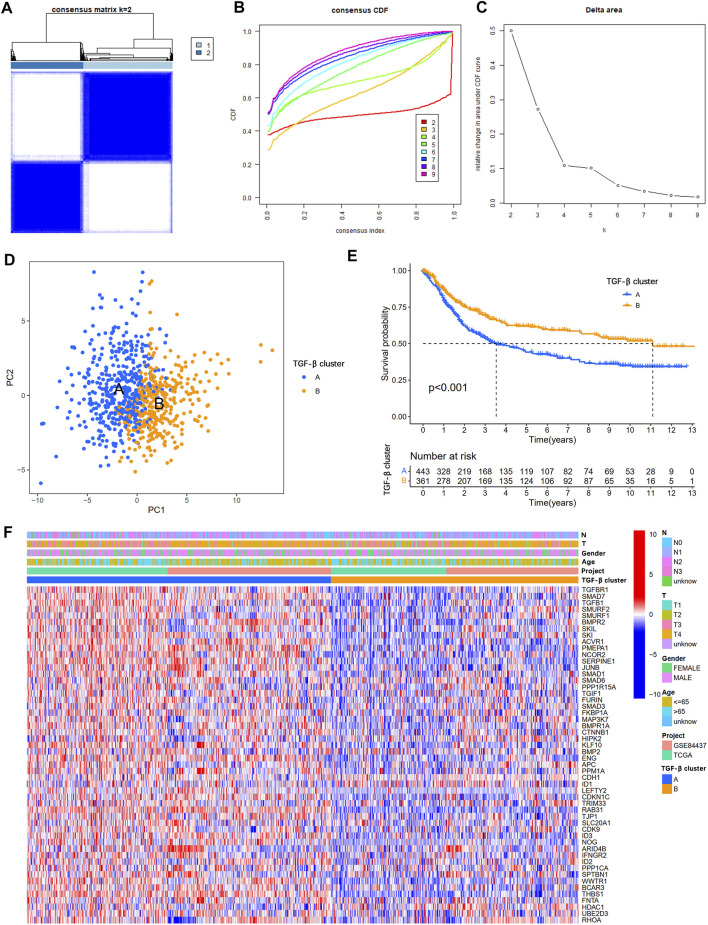
Overall survival and clinicopathological characteristics of two different TSRG subgroups. **(A)** Consensus matrix heatmap defining two clusters (*k* = 2). **(B)** The cumulative distribution function (CDF) from *k* = 2 to 9. **(C)** Relative variation of the area under the CDF region at *k* = 2–9. **(D)** PCA shows different distributions between the two subgroups. **(E)** Kaplan-Meier survival analysis between two different TSRG subgroups. **(F)** Differences in clinicopathologic characteristics and expression levels of TSRGs between the two distinct TSRG subgroups. TSRGs, TGF-β signaling related genes; CDF, cumulative distribution function; PCA, principal components analysis.

To understand the expression pattern of TSRGs involved in tumorigenesis, data from 804 gastric cancer samples from TCGA-STAD and GSE84437 datasets were enrolled in our study for further analysis (Supplementary Table S2). To explore the characteristics of 54 TSRGs expression profiles in gastric cancer, we performed unsupervised clustering analysis to identify gastric cancer subtypes based on 54 TSRGs expression levels. The results showed that *K* = 2 was the most appropriate cluster, and 804 gastric cancer patients were classified into TGF-β cluster A (*n* = 443) and TGF-β cluster B (*n* = 361) (Figures 2A–C and Supplementary Table S3). The PCA results further demonstrate the excellent grouping effect (Figure 2D). Kaplan-Meier survival analysis showed a more significant survival advantage for TGF-β cluster B (*p* < 0.001, Figure 2E). In addition, we combined TGF-β subgroups and clinicopathological features of gastric cancer patients to map 54 TSRGs expression heatmaps and found that 54 TSRGs were expressed at higher levels in TGF-β cluster A compared to TGF-β cluster B (Figure 2F).

### Characteristics of the TME in two distinct TGF-β subgroups

**FIGURE 3:**
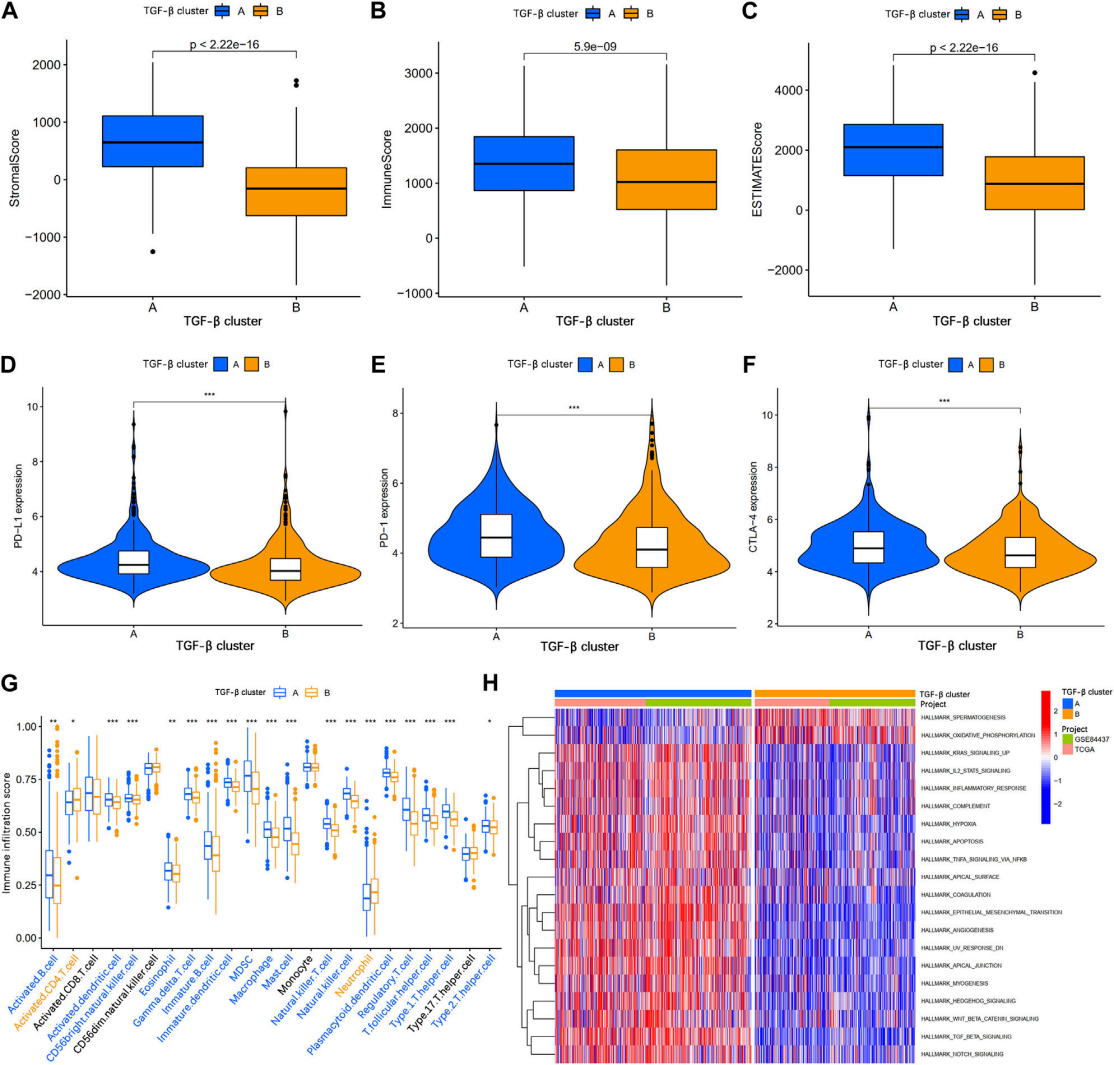
Analysis of the tumor immune microenvironment between two different TGF-β subgroups. **(A–C)** Stromal score, immune score, and ESTIMATE score analyses between two subgroups. **(D–F)** Expression levels of PD-1, PD-L1, and CTLA-4 in the two subgroups. **(G)** The abundance of 23 infiltrating immune cell types in the two different TGF-β subgroups. **(H)** GSVA of biological pathways between two subgroups. GSVA, gene set variation analysis; PD-1, programmed cell death 1; PD-L1, programmed cell death 1 ligand 1; CTLA-4, cytotoxic T-lymphocyte associated protein 4; *p < 0.05; **p < 0.01; ***p < 0.001.

To explore the correlation between TSRGs and TME in gastric cancer, we first performed an ESTIMATE analysis. The results showed that patients in TGF-β cluster A had a higher stromal score, immune score, and ESTIMATE score (Figures 3A–C), suggesting that gastric cancer patients in the TGF-β cluster A have higher immune activity and lower tumor purity. Then, expression analysis of three crucial immune checkpoint genes (PD1, PD-L1, and CTLA4) showed higher expression levels of PD1, PD-L1, and CTLA4 in gastric patients in the TGF-β cluster A compared to patients in TGF-β cluster B (Figures 3D–F). We further analyzed the level of infiltration of 23 immune cells in patients with two distinct TGF-β clusters using the CIBERSORT algorithm. As shown in Figure 3G, the infiltration levels of activated B cell, activated dendritic cell, CD56 bright natural killer cell, eosinophil, gamma delta T cell, immature B cell, immature dendritic cell, MDSC, macrophage, mast cell, natural killer T cell, natural killer cell, plasmacytoid dendritic cell, regulatory T cell, T follicular helper cell, type 1 T helper cell, and type 2 T helper cell were higher in the TGF-β cluster A than those in the TGF-β cluster B, while activated CD4 T cell and neutrophil had significantly lower infiltration in TGF-β cluster A than those in the TGF-β cluster B. In addition, GSVA enrichment analysis revealed multiple tumor-associated signaling pathways enriched in TGF-β cluster A, including KRAS, IL2/STAT5, inflammatory response, hypoxia, apoptosis, and wnt/β-catenin signaling pathways (Figure 3H).

### Identification of gene clusters based on TGF-β pattern-related DEGs

**FIGURE 4:**
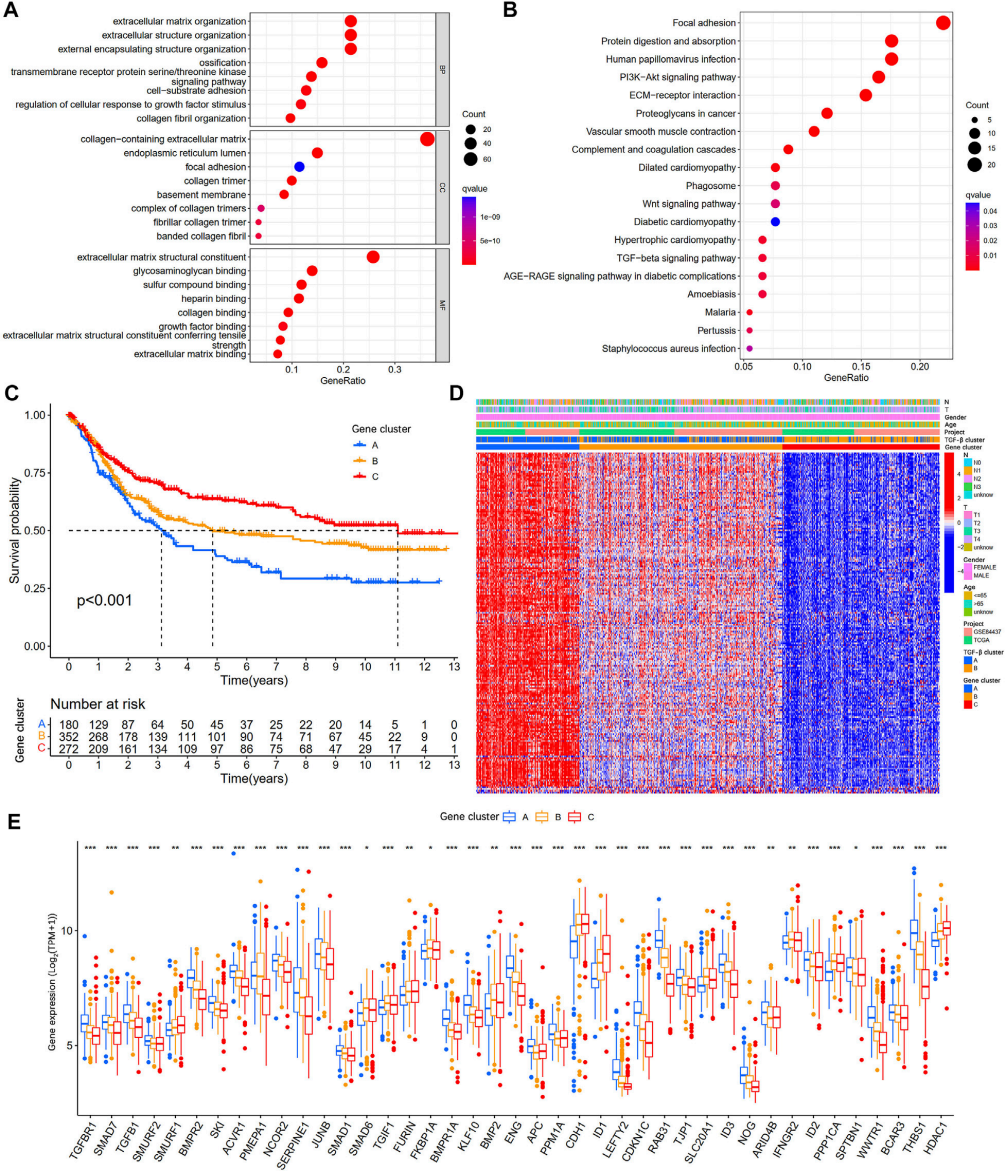
Identification of gene subgroups based on DEGs among two TGF-β subgroups. **(A,B)** GO and KEGG enrichment analyses of DEGs among two TGF-β subgroups. **(C)** Kaplan-Meier survival analysis between three different gene subgroups. **(D)** Heatmap of clinicopathologic characteristics and DEGs expressions among the three gene subgroups. **(E)** Differences in the expression of 54 TSRGs among the three gene subgroups. DEGs, differentially expressed genes; GO, Gene Ontology; KEGG, Kyoto Encyclopedia of Genes and Genomes; TSRGs, TGF-β signaling related genes; *p < 0.05; **p < 0.01; ***p < 0.001.

To further explore the potential biological functions of the TGF-β clusters, we obtained 202 TGF-β clusters-related DEGs (Supplementary Table S4) using R package limma and performed functional enrichment analysis. These TGF-β cluster-related DEGs are mainly enriched in biological processes associated with the extracellular matrix (Figure 4A). KEGG analysis showed that DEGs were associated with metastasis and tumor-related signaling pathways (Figure 4B), suggesting that TSRGs play an essential role in tumorigenesis and metastasis. Then, 202 TGF-β cluster-related DEGs were subjected to univariate Cox regression analysis to screen for genes associated with OS in gastric cancer. We obtained 199 genes related to the prognosis of gastric cancer patients at *p* < 0.05 (Supplementary Table S5). To further explore the potential mechanisms of prognosis-related DEGs in gastric cancer, based on the expression level of 199 prognostic genes, unsupervised consensus clustering analysis was utilized to classify gastric cancer patients into three different gene clusters, namely gene cluster A, gene cluster B, and gene cluster C (Supplementary Table S6). Kaplan-Meier survival analysis showed that patients in gene cluster A had the worst OS, whereas patients in gene cluster C showed a superior OS (Figure 4C). In addition, we combined the TGF-β cluster, gene cluster, and clinicopathological features of gastric cancer patients to map heat maps and found significant expression differences among gene clusters (Figure 4D). The three gene clusters showed significance in TSRGs expression, as expected from the TGF-β clusters (Figure 4E).

### Construction and validation of the risk model

**FIGURE 5:**
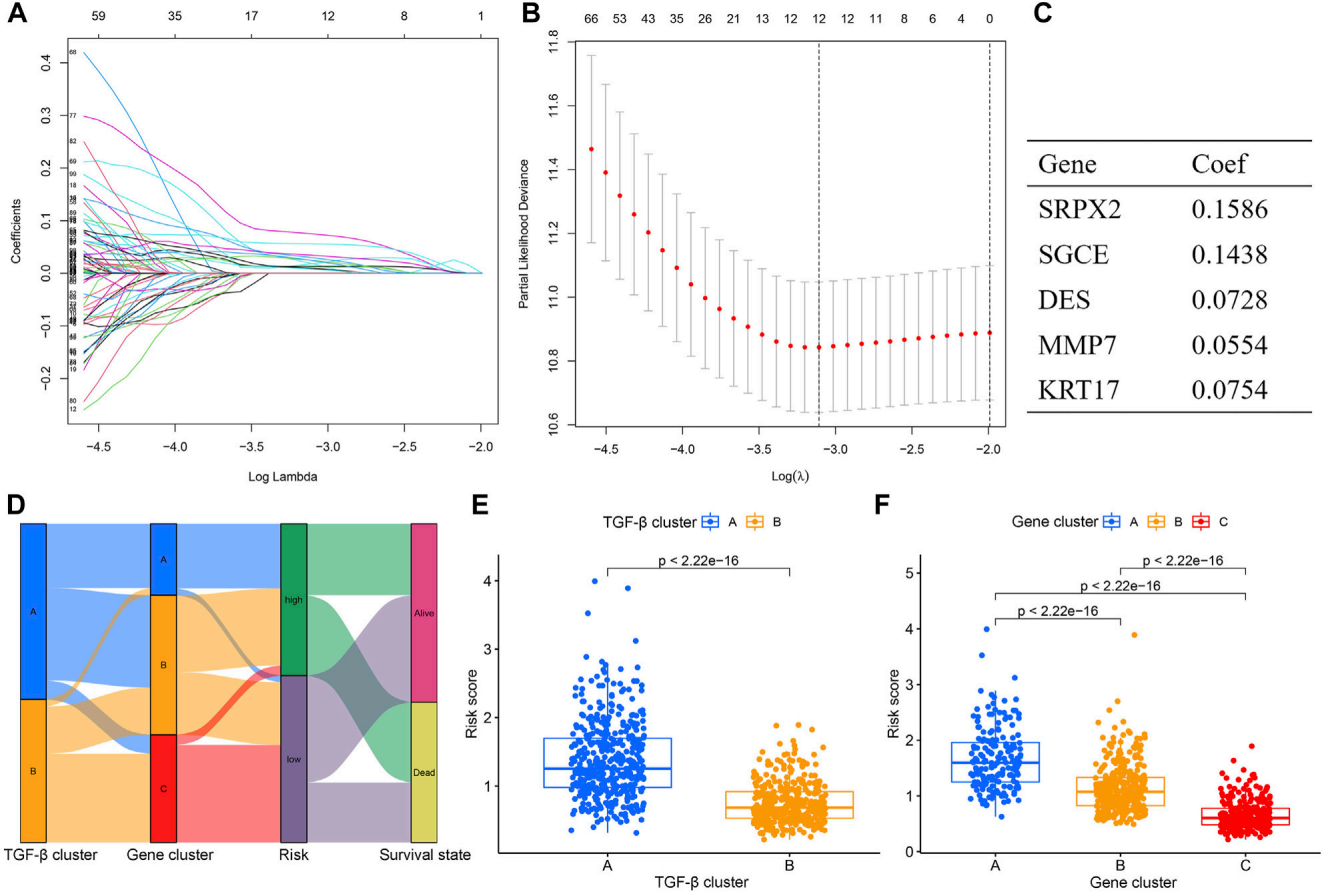
Construction of the TGF-β cluster-related DEGs prognostic model. **(A,B)** Twelve optimal TGF-β cluster-related DEGs were found using the LASSO cox regression. **(C)** Five optimal TGF-β cluster-related DEGs were found using the multivariate Cox analysis. **(D)** Sankey diagram of TGF-β cluster, gene cluster, risk score, and survival status. **(E)** Differences in risk score between two TGF-β clusters. **(F)** Differences in risk score between three gene clusters. DEGs, differentially expressed genes; LASSO, least absolute shrinkage and selection operator; Coef, coefficient.

To quantify the risk of each gastric cancer patient, we constructed a prognostic risk model based on TGF-β cluster-related prognostic DEGs. First, the R package caret was used to randomize patients into a training set (*n* = 402) (Supplementary Table S7). And a testing set (*n* = 402) (Supplementary Table S8) at a ratio of 1:1. Second, in the training set, LASSO and multivariate Cox regression analyses were used to construct an appropriate risk model. Based on the minimum partial likelihood deviance, 12 potential candidate genes were screened by LASSO regression analysis (Figures 5A,B; Supplementary Table S9). Subsequent multivariate Cox regression of 12 prognosis-related genes yielded five genes used to construct the risk model, namely SRPX2, SGCE, DES, MMP7, and KRT17. We calculated the risk score for each patient based on the formula. Risk score= (0.1586×expression of SRPX2) + (0.1438×expression of SGCE) + (0.0728×expression of DES) + (0.0554×expression of MMP7) + (0.0754×expression of KRT17) (Figure 5C). The Sankey diagram showed the correlation between risk score and TGF-β clusters, gene clusters, and survival status (Figure 5D). In addition, we observed an obvious difference in the risk score of the TGF-β clusters and gene clusters (Figures 5E,F). The previous survival analysis showed shorter OS in the TGF-β cluster A and gene cluster A groups, and our model showed the highest risk scores in TGF-β cluster A and gene cluster A groups, which further demonstrated the excellent performance of our risk model.

**FIGURE 6:**
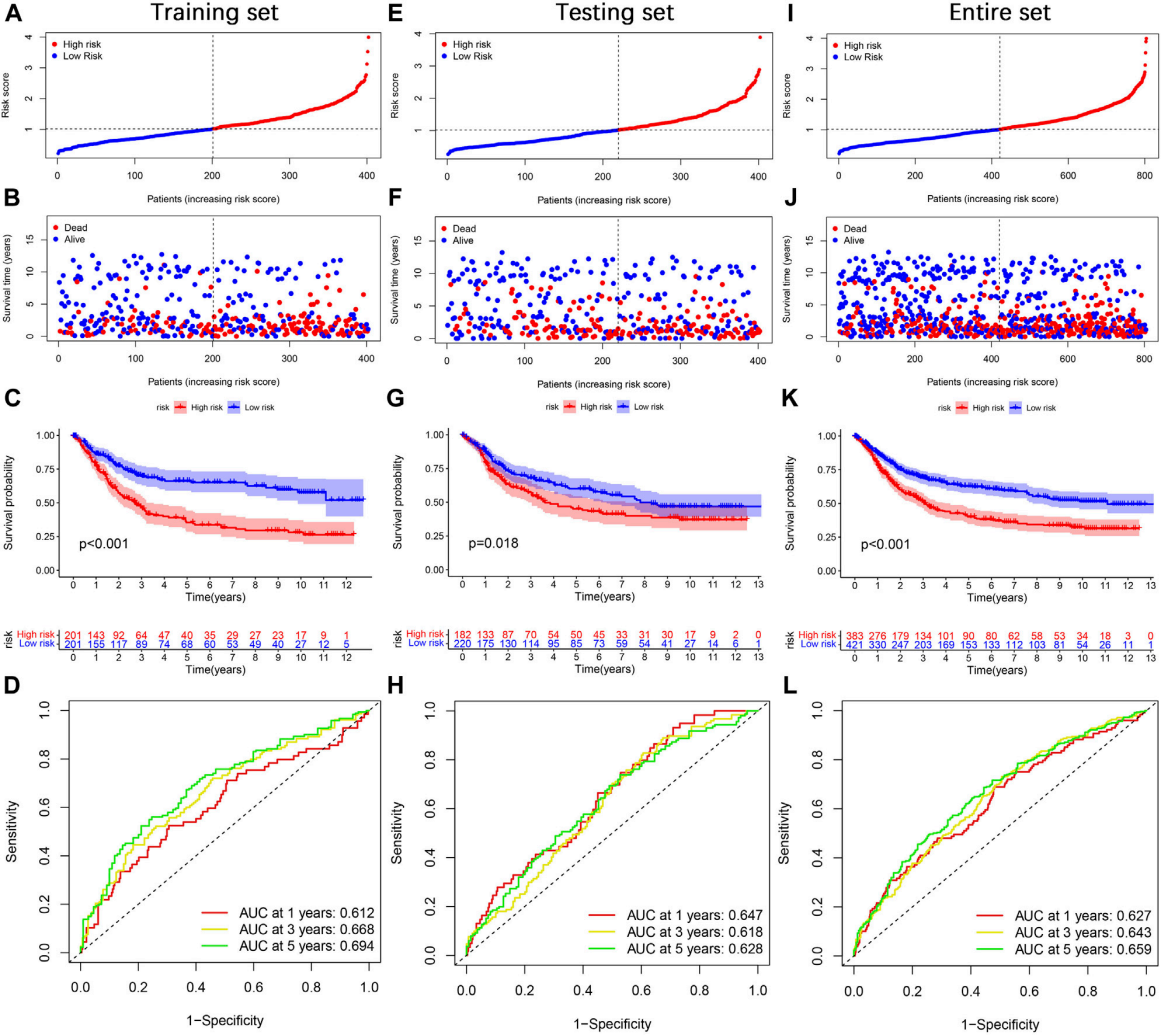
Prognosis value of the TGF-β cluster-related DEGs prognostic model. **(A)** The distribution of risk score, **(B)** survival status, **(C)** Kaplan–Meier survival curves, **(D)** the 1-, 3-, and 5-year ROC curves. **(E–L)** The validation sets, including the testing set and the entire set, were analyzed similarly.

Next, we divided gastric cancer patients into high- and low-risk groups based on the median risk score. The risk score curve and survival status scatter plots show that the number of deaths in gastric cancer patients increases as the risk score increases (Figures 6A,B). Kaplan-Meier survival analysis showed that patients in the high-risk group had worse OS than those in the low-risk group (Figure 6C). The risk score’s 1-, 3-, and 5-year AUC values were 0.612, 0.668, and 0.694, respectively (Figure 6D). Meanwhile, we did the same analysis in two validation sets (the testing set and the entire set), respectively, and we obtained similar results (Figures 6E–L). In the IMvigor210 cohort, patients in the high-risk group had significantly lower survival than the low-risk group (Supplementary Figure S2A), which further validates the accuracy of our constructed prognostic model. Taken together, our established risk model has an excellent performance in predicting the survival outcome of gastric cancer patients.

### Clinical correlation analysis and stratification analysis of the risk model

**FIGURE 7:**
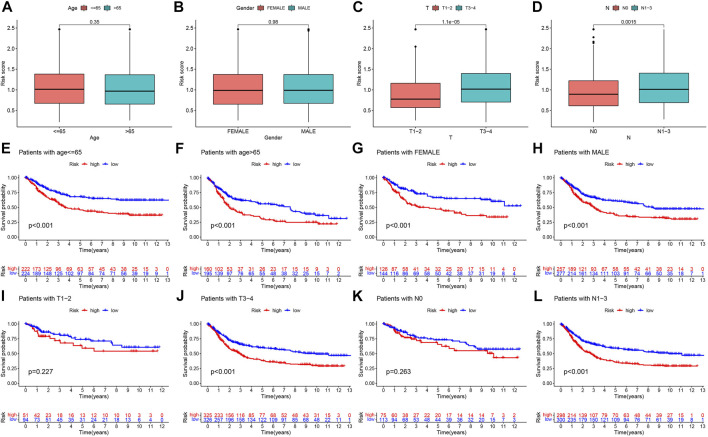
Prognostic model-based clinicopathological characteristics and survival subgroup analysis, Differential analysis of the risk score for **(A)** age, **(B)** gender, **(C)** T stage, and **(D)** N stage subgroups. Kaplan-Meier survival analysis for **(E)** age ≤65 years, **(F)** age >65 years, **(G)** female, **(H)** male, **(I)** T1-2, **(J)** T3-4, **(K)** N0, and **(L)** N1-3 between high- and low-risk groups.

To explore the correlation between the risk score and available clinicopathological characteristics, we first analyzed differences in risk scores across clinical subgroups. The subgroups were divided by age (≤65 years or >65 years), sex (female or male), T stage (T1-2 or T3-4), and N stage (N0 or N1-3). The results showed that the risk scores were not statistically different across age and gender subgroups (Figures 7A,B), while patients in the T3-4 and N1-3 subgroups had higher risk scores (Figures 7C,D). In addition, we performed Kaplan-Meier survival analysis for different subgroups. We found that in the age ≤65 years (Figure 7E), age >65 years (Figure 7F), female (Figure 7G), male (Figure 7H), T3-4 (Figure 7J), N1-3 (Figure 7L) subgroups of gastric cancer patients, the OS of patients in the high-risk group was significantly lower than that of low-risk patients, while no significant differences were seen for T1-2 (Figure 7I), N0 (Figure 7K) subgroups.

### Independent prognostic and nomogram analysis

**FIGURE 8:**
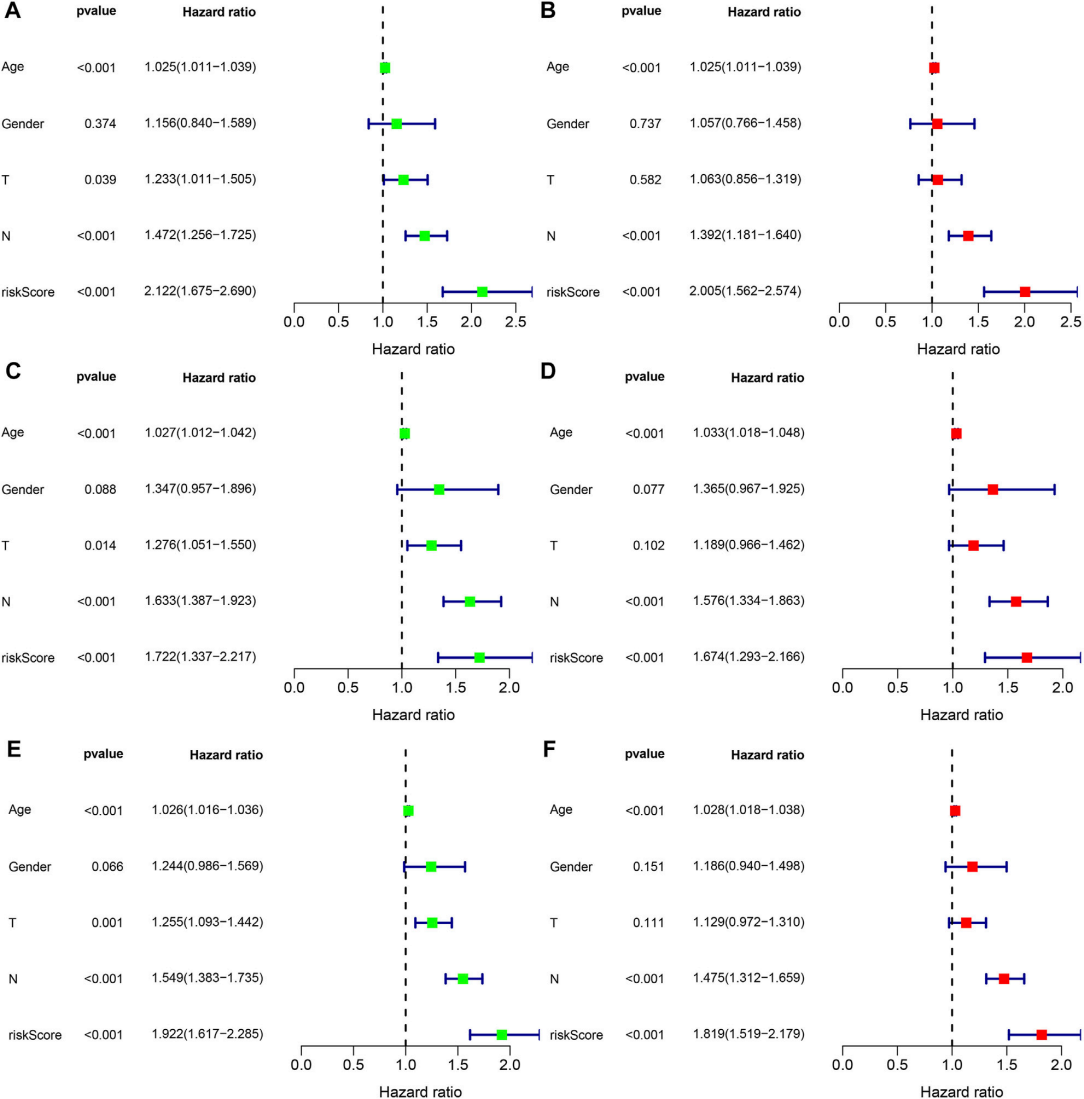
The independent prognosis analysis of the risk score and clinicopathological variables in gastric cancer. **(A,B)** Univariate and multivariate Cox regression analyses of clinicopathological variables and risk scores with OS in the training set, **(C,D)** testing set, and **(E,F)** entire set.

To explore whether the risk score is an independent prognostic factor for patients with gastric cancer, we performed univariate and multivariate Cox regression analyses in the training set and two validation sets (testing set and entire set) in combination with clinicopathological characteristics. In the training set, univariate Cox regression analysis displayed that age (HR = 1.025, 1.011–1.039, *p* < 0.001), T stage (HR = 1.233, 1.011–1.505, *p* = 0.039), N stage (HR = 1.472, 1.256–1.725, *p* < 0.001), and risk score (HR = 2.122, 1.675–2.690, *p* < 0.001) predicted worse OS (Figure 8A). Multivariate Cox regression analysis showed that the age (HR = 1.028, 1.018–1.038, *p* < 0.001), N stage (HR = 1.392, 1.181–1.604, *p* < 0.001) and risk score (HR = 2.005, 1.562–2.574, *p* < 0.001) were independent prognostic factors in gastric cancer patients (Figure 8B). In the testing set, univariate Cox regression analysis displayed that age (HR = 1.027, 1.012–1.042, *p* < 0.001), T stage (HR = 1.276, 1.051–1.550, *p* = 0.014), N stage (HR = 1.633, 1.387–1.923, *p* < 0.001), and risk score (HR = 1.722, 1.337–2.217, *p* < 0.001) predicted worse OS (Figure 8C). Multivariate Cox regression analysis showed that the age (HR = 1.033, 1.018–1.048, *p* < 0.001), N stage (HR = 1.576, 1.334–1.863, *p* < 0.001) and risk score (HR = 1.674, 1.293–2.166, *p* < 0.001) were independent prognostic factors in gastric cancer patients (Figure 8D). In the entire set, univariate Cox regression analysis displayed that age (HR = 1.026, 1.016–1.036, *p* < 0.001), T stage (HR = 1.255, 1.093–1.442, *p* = 0.001), N stage (HR = 1.549, 1.383–1.735, *p* < 0.001), and risk score (HR = 1.922, 1.617–2.285, *p* < 0.001) predicted worse OS (Figure 8E). Multivariate Cox regression analysis showed that the age (HR = 1.028, 1.018–1.038, *p* < 0.001), N stage (HR = 1.475, 1.312–1.659, *p* < 0.001) and risk score (HR = 1.819, 1.519–2.179, *p* < 0.001) were independent prognostic factors in gastric cancer patients (Figure 8F). Taken together, the risk score is an independent prognostic factor for patients with gastric cancer.

**FIGURE 9:**
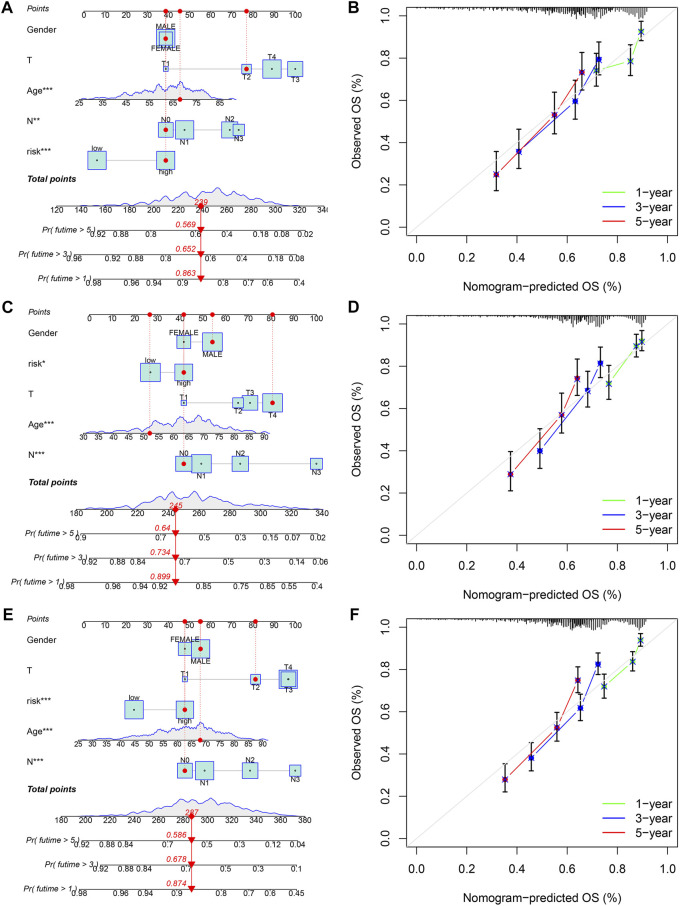
Construction and validation of a nomogram for predicting OS in gastric cancer. **(A,B)** The nomogram combining gender, age, T stage, N stage, and risk score for predicting gastric cancer patient OS at 1-, 3-, and 5- years in the training set, **(C,D)** testing set, and **(E,F)** entire set. *p < 0.05; **p < 0.01; ***p < 0.001.

Given the close correlation between the risk score and prognosis of gastric cancer patients, we integrated gender, age, T stage, N stage, and risk score to plot a nomogram to predict the 1-, 3-, and 5-year survival rates in the training set and two validation sets (testing set and entire set) (Figures 9A,C,E). Furthermore, the 1-, 3-, and 5-year calibration curves showed great accuracy between the nomogram-predicted OS and the actual observed OS (Figures 9B,D,F).

### Analysis of tumor immune microenvironment between high- and low-risk groups

**FIGURE 10:**
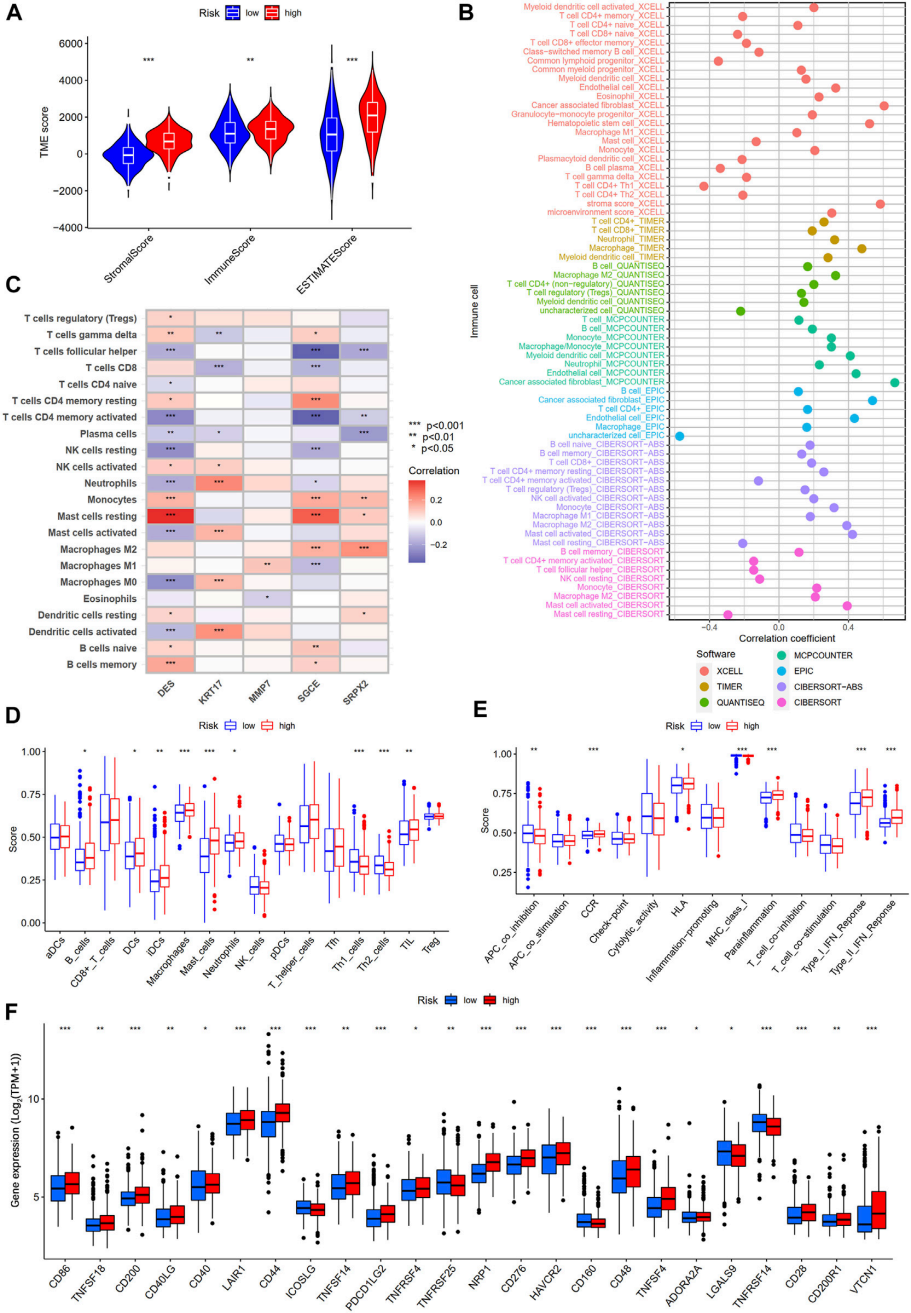
Differential analysis of tumor immune microenvironment between high- and low-risk groups. **(A)** TME score between high- and low-risk groups. **(B)** Spearman correlation analysis of immune components and risk scores based on XCELL, TIMER, QUANTISEQ, MCPCOUNTER, EPIC, CIBERSORT-ABS, and CIBERSORT algorithms. **(C)** Spearman correlations between the abundance of immune cells and five genes in the prognostic model. **(D)** 16 immune cells and **(E)** 13 immune-related functions between the high- and low-risk groups by ssGSEA. **(F)** The expression of immune checkpoint-related genes between the high- and low-risk groups.

To explore the differences in tumor immune microenvironment between high- and low-risk groups of gastric cancer patients, we first performed ESTIMATE analysis. The results showed that gastric patients in the high-risk group had a higher stromal score, immune score, and ESTIMATE score (Figure 10A). Subsequently, seven algorithms were used to assess the correlation between the level of immune cell infiltration and the risk score. As shown in Figure 10B, the risk score was positively correlated with myeloid dendritic cell, CD4^+^ T cell, CD8^+^ T cell, cancer-associated fibroblast, hematopoietic stem cell, neutrophil, and macrophage M2, while negatively correlated with T cell CD4^+^ memory activated, T cell follicular helper, NK cell resting, and mast cell resting (Supplementary Table S10). We also performed a correlation analysis between the five genes in our prognostic model and the immune cells. We found that DES, KRT17, SGCE, and SRPX2 were significantly correlated with most immune cells, while MMP7 only correlated with macrophages M1 and eosinophils (Figure 10C). In addition, we further explored the difference of 16 immune cells and 13 immune-related pathways between the two subgroups using ssGSEA. We found that B cells, DCs, iDCs, macrophages, mast cells, neutrophils, TIL, CCR, HLA, parainflammation, type I IFN response, and type II IFN response were more enriched in the high-risk group, while the Th1 cells, Th2 cells, APC co inhibition, and MHC class I is less enriched in the high-risk group (Figures 10D,E). Finally, we analyzed the expression levels of immune checkpoint-related genes between two subgroups. Figure 10F showed that 24 immune checkpoint-related genes were differentially expressed in the high- and low-risk groups.

### Immunotherapy response analysis

**FIGURE 11:**
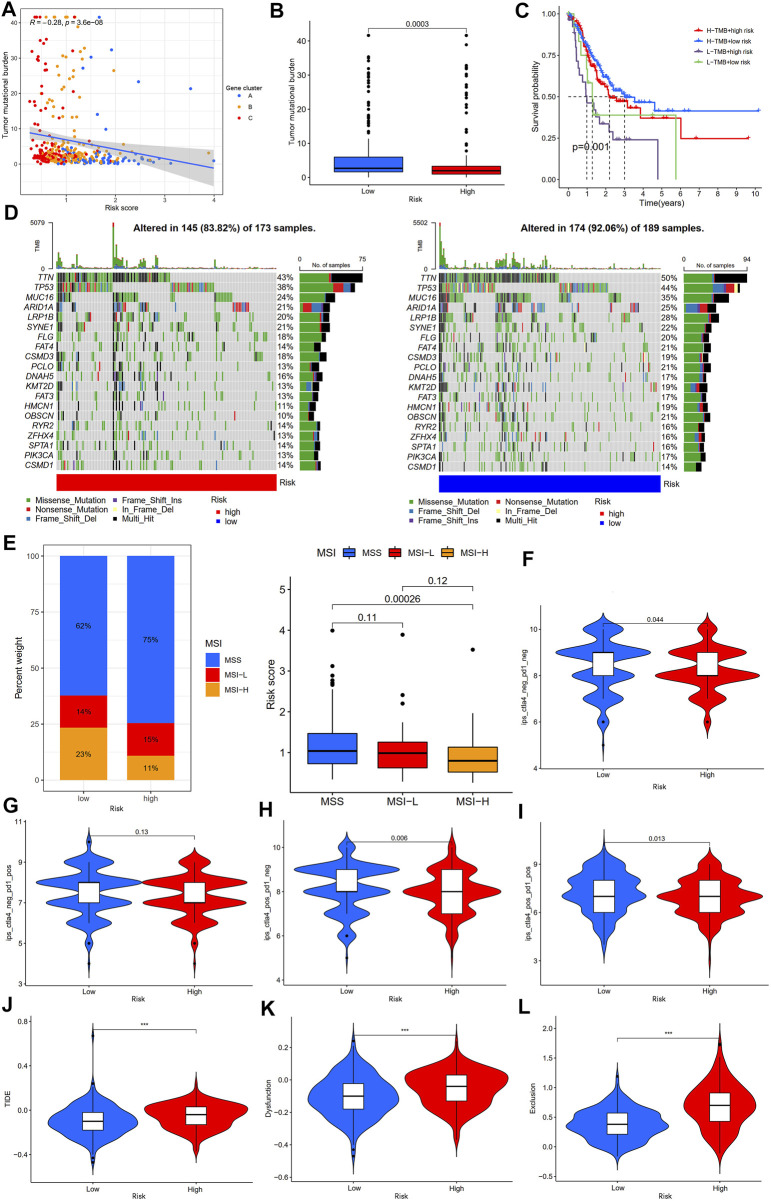
Analysis of immunotherapy response rates between high- and low-risk groups. **(A)** Spearman correlation analysis of the risk score and TMB. **(B)** Analysis of TMB differences between high- and low-risk groups. **(C)** Kaplan-Meier survival analysis among four subgroups stratified by both TMB and risk score. **(D)** The waterfall plot of somatic mutation landscape high- and low-risk groups. **(E)** Relationships between risk score and MSI. **(F)** The ips_ctla4_neg_pd1_neg, **(G)** ips_ctla4_neg_pd1_pos, **(H)** ips_ctla4_pos_pd1_neg, and **(I)** ips_ctla4_pos_pd1_pos analyses between the high- and low-risk groups. **(J–L)** The TIDE, dysfunction, and exclusion score analyses between the high- and low-risk groups. TMB, tumor mutation burden; IPS, immunophenoscore; TIDE, tumor immune dysfunction and exclusion; ***p < 0.001.

TMB and MSI are considered biomarkers of tumor immunotherapy response rate (Rizzo et al., 2021), and patients with high TMB and MSI-H benefit from immunotherapy and have more prolonged survival. Therefore, we first analyzed the correlation between the TMB and risk score. The results showed a negative correlation between the TMB and risk score (Figure 11A), and the TMB of gastric cancer patients in the low-risk group was significantly higher than that of gastric cancer patients in the high-risk group (Figure 11B). Kaplan-Meier survival analysis showed that the risk score diminished the prognostic advantage of patients with gastric cancer in the high TMB group (Figure 11C). We further analyzed the somatic mutations in the high- and low-risk groups of gastric cancer patients. The results showed that the most common form of mutation was missense mutation, and the top five mutated genes were TTN, TP53, MUC16, ARID1A, and LRP1B, and the frequency of mutations was higher in the low-risk group (Figure 11D), which was consistent with the results of the above study. In addition, we analyzed the correlation between the MSI and risk scores and showed that patients in the low-risk group had a higher proportion of MSI-H and that patients with MSI-H had lower risk scores (Figure 11E).

IPS and TIDE scores are novel tumor immunotherapy response rate biomarkers that better assess the efficacy of anti-PD1 and anti-CTLA4 therapies. A high IPS score represents higher immunogenicity, and a high TIDE score represents a greater likelihood of tumor immune escape (Wu et al., 2021; Zeng et al., 2022); therefore, the higher the IPS and the lower the TIDE score, the better the patient’s outcome to immunotherapy. Our results showed that gastric cancer patients in the low-risk group had higher IPS (Ips_ctla4_neg_pd1_neg, ips_ctla4_pos_pd1_neg, and ips_ctla4_pos_pd1_pos scores) than those in the high-risk group, but there was no statistically significant difference between the two groups in the ips_ctla4_neg_pd1_pos score (Figures 11F–I). Furthermore, the TIDE, dysfunction, and exclusion scores of gastric cancer patients in the low-risk group were lower than those in the high-risk group (Figures 11J–L). In addition, analysis of immunotherapy response based on the IMvigor210 cohort showed that patients in the immunotherapy-responsive group (complete response (CR)/partial response (PR) group) had significantly lower risk scores than the immunotherapy non-responsive group (stable disease (SD)/progressive disease (PD) group) (Supplementary Figure S2B). The above results suggest that patients with gastric cancer in the low-risk group may be may be more sensitive to immunotherapy.

### Antitumor drug sensitivity analysis

**FIGURE 12:**
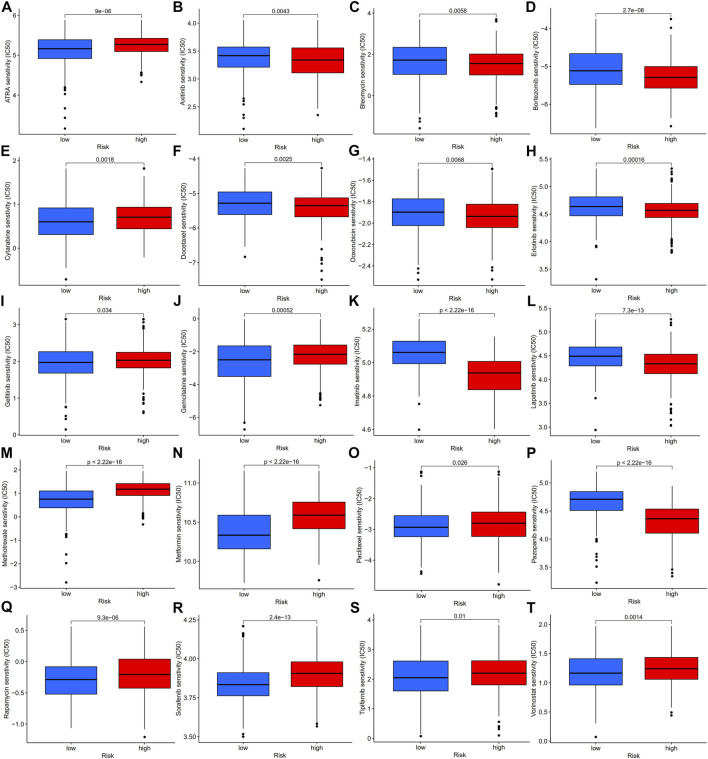
Antitumor drug sensitivity analysis of gastric patients in high- and low-risk groups, IC50 analysis of ATRA **(A)**, axitinib **(B)**, bleomycin **(C)**, bortezomib **(D)**, cytarabine **(E)**, docetaxel **(F)**, doxorubicin **(G)**, erlotinib **(H)**, gefitinib **(I)**, gemcitabine **(J)**, imatinib **(K)**, lapatinib **(L)**, methotrexate **(M)**, metformin **(N)**, paclitaxel **(O)**, pazopanib **(P)**, rapamycin **(Q)**, sorafenib **(R)**, tipifarnib **(S)**, and vorinostat **(T)** in the high- and low-risk groups, which were classified by the prognostic model. IC50, half-maximal inhibitory concentration.

To explore the potential role of our established risk model for clinical treatment, we analyzed the differences in IC50 of common antitumor drugs between high- and low-risk groups. We found that gastric cancer patients in the low-risk group were more sensitive to ATRA, cytarabine, gefitinib, gemcitabine, methotrexate, metformin, paclitaxel, rapamycin, sorafenib, tipifarnib, and vorinostat than those in the high-risk group, while gastric cancer patients in the low-risk group were less sensitive to axitinib, bleomycin, bortezomib, docetaxel, doxorubicin, erlotinib, imatinib, lapatinib, and pazopanib than those in the high-risk group (Figure 12). The above results suggest that our prognostic model can be an essential indicator of antitumor drugs for patients with gastric cancer.

## Discussion

Numerous studies have shown that the TGF-β signaling pathway plays an essential role in the tumor immune microenvironment and can exert both pro- and anti-tumor effects (Morikawa et al., 2016; Colak and Ten Dijke, 2017; Garcia-Rendueles et al., 2017; Batlle and Massagué, 2019; Kim et al., 2021). However, most studies focus on one or two TGF-β signaling pathway genes or a single TME cell, and the overall TME infiltration characteristics mediated by the multiple TGF-β signaling pathway genes have not been comprehensively understood. Discovering the role of different TGF-β-related subtypes in the TME will help improve our understanding of the antitumor immune microenvironment and guide more precise individualized immune therapy.

In this study, we first analyzed the differential expression levels and genetic mutation characteristics of 54 TSRGs using the TCGA-STAD dataset. Although the mutation frequency of 54 TSRGs was low, most were highly expressed and closely related in gastric cancer. Subsequently, we identified two distinct TGF-β subgroups, TGF-β cluster A and TGF-β cluster B, based on 54 TSRGs transcriptome expression levels using an unsupervised clustering approach. Compared to gastric cancer patients with TGF-β cluster B, gastric cancer patients with TGF-β cluster A had shorter OS, higher expression levels of 54 TSRGs, higher stromal scores, immune scores, ESTIMATE scores, higher levels of PD1, PD-L1, CTLA4 expression levels, and higher infiltration levels of MDSC, macrophage, and regulatory T cells. The above results imply that TGF-β cluster A has a more active immunosuppressive TME. Tumor cells in the immunosuppressive TME can evade the killing effect of immune cells and have a high degree of malignancy, which in turn leads to a shorter survival of patients (Lei et al., 2020). And the patients with TGF-β cluster A in this study had shorter survival, which is consistent with this phenomenon. Next, we identified the DEGs between two distinct TGF-β subgroups and further identified three gene subgroups based on DEGs. There was a significant difference in OS between the three gene subgroups. In addition, 41 of the 54 TSRGs were significantly differentially expressed among the three gene subgroups. This demonstrated a close association between gene subgroups and TGF-β subgroups.

Next, we constructed a TGF-β-related prognostic model to calculate the risk score for each patient. We first screened prognosis-related genes by univariate Cox regression analysis for differentially expressed genes between two TGF-β subgroups. Next, LASSO Cox regression analysis was used to construct a prognostic model containing five genes, and each patient’s risk score was calculated. We found that TGF-β cluster A and gene cluster A were mainly concentrated in the high-risk group, while TGF-β cluster B and gene cluster C were primarily concentrated in the low-risk group. Patients in the high-risk group had a poor prognosis, consistent with the previous results of poor prognosis in the TGF-β cluster A and gene cluster A groups. The five genes in the prognostic model were SRPX2, SGCE, DES, MMP7, and KRT17. Studies have shown that SRPX2 is highly expressed in gastric cancer and can promote migration and adhesion of gastric cancer cells, which is closely associated with poor prognosis of gastric cancer patients (Tanaka et al., 2009). The present study showed that SRPX2 is a risk factor for the prognosis of gastric cancer patients, which is consistent with the above findings. SGCE has a hazard ratio greater than 1 in gastric cancer and is considered a poor prognostic marker (Hou et al., 2017), which is consistent with the results of this study. SGCE is a sponge molecule of EGFR and its E3 ubiquitination ligase (c-Cbl). High expression of SGCE inhibits EGFR degradation *via* the ubiquitin lysosomal pathway, increases tumor cell drug resistance, and promotes metastasis (Zhao et al., 2020b). Studies have shown that desmin (DES) protein is more advantageous than elastin protein in detecting vascular invasion in gastric cancer and is considered one of the markers of tumor invasion (Ekinci et al., 2018; Shin et al., 2020). MMP7 expression was significantly associated with poor clinicopathological features of gastric cancer patients, including vascular invasion, undifferentiated histological types, higher TNM stage, and high CEA levels (Wattanawongdon et al., 2022), and was considered one of the prognostic markers of gastric cancer (Chang et al., 2014). It was shown that silencing KRT inhibited the proliferation, migration, and invasion of gastric cancer cells, induced apoptosis, and stalled the gastric cancer cell cycle at the G1/S phase by decreasing the expression of cyclin E1 and cyclin D (Hu et al., 2018). In addition, Zhou et al. constructed a prognostic signature based on multiple gastric cancer datasets in the GEO database, including MMP7 and KRT17 (Zhou et al., 2021), which indirectly demonstrated the reliability of our prognostic model. Next, we performed a survival analysis between high- and low-risk groups, which showed that OS was worse in the high-risk group of gastric cancer patients. This result was also confirmed in both validation sets (testing set and entire set). The risk scores also had excellent performance across clinicopathological characteristics subgroups. Univariate and multifactorial Cox regression analyses demonstrated that the risk score was an independent prognostic factor for patients with gastric cancer. In addition, the nomograms constructed by the risk score combined with clinicopathological characteristics also excelled in predicting the overall survival of gastric cancer patients at 1-, 3-, and 5-year. Overall, the TGF-β-related prognostic model we constructed could excellently predict the prognosis of gastric cancer patients.

The TME is the internal environment on which tumor cells depend for survival. Under normal circumstances, immune cells in the TME can recognize and remove tumor cells on time, but tumor cells can create an immunosuppressive TME through a complex regulatory network to produce immune escape (Joyce and Fearon, 2015; Jiang et al., 2019). The immunosuppressive TME consists of immunosuppressive cells such as regulatory T cells (Tregs), tumor-associated macrophages (TAMs), tumor-associated neutrophils (TANs), myeloid-derived suppressor cells (MDSCs), tumor-associated fibroblasts (CAFs), extracellular matrix, suppressive cytokines such as interleukin 10, interleukin 17, TGF-β exosomes and immune checkpoint molecules such as PD1, PD-L1, and CTLA4 (Zhang et al., 2019; Li et al., 2020; Nakamura and Smyth, 2020). Studies have shown that increased MDSCs in tumor tissues promote the production of Tregs and deplete activated T cells (Davis et al., 2016). Furthermore, Tregs can inhibit CD80 and CD86 co-stimulatory signaling *via* CTLA4, secrete suppressive cytokines, and kill effector T cells (Tekguc et al., 2021). TAMs can enhance the immunosuppressive TME in several ways. In gastric cancer, TAMs promote PD-L1 expression through the secretion of CXCL8, thereby suppressing the antitumor effects of CD8^+^ T cells (Lin et al., 2019). TAMs can also recruit Tregs through the secretion of chemokines such as CCL2, CCL3, CCL20, and CCL22, which in turn form immunosuppressive TMEs(Cassetta and Pollard, 2020; Pan et al., 2020). In addition, TAMs-derived TGF-β can promote its secretion of CCL22 to recruit Tregs, which in turn can secrete IL-8 to promote TGF-β secretion by TAMs, thereby enhancing immunosuppressive TME (Wang et al., 2019). This study showed higher MDSC, macrophage, and regulatory T cell infiltration levels and more active signaling pathways such as TGF-β, and Wnt/β-catenin signaling pathways in the TGF-β cluster A, suggesting a more active immunosuppressive microenvironment. Spearman correlation analysis of immune cells and risk scores showed a positive correlation between risk scores and myeloid dendritic cells, M2 macrophages, and CAFs, suggesting that the TME of patients in the high-risk group was immunosuppressive. Patients with gastric cancer of TGF-β cluster A were mainly concentrated in the high-risk group, and the results of the before-and-after study were consistent.

ICIs offer new hope for patients with advanced cancer due to their significant efficacy and fewer side effects. However, only a small number of patients can benefit from them. Therefore, there is an urgent need to screen the population with a high response rate for more precise treatment. Currently, common biomarkers to predict the efficacy of ICIs include TMB, microsatellite status, IPS, and TIDE score. Tumor cells with MSI-H have an increased TMB and generate new antigens due to unrepaired mis-replicated DNA, which allows more TILs to infiltrate and thus respond better to ICIs (Lizardo et al., 2020). This study showed that the low-risk group had a higher TMB and a higher percentage of MSI-H than the high-risk group, suggesting that low-risk gastric cancer patients may have a better treatment effect on ICIs. IPS and TIDE scores are novel immunotherapy biomarkers with good predictive power for response rates to ICIs (Wu et al., 2021). A high IPS represents higher immunogenicity, and a high TIDE score represents a greater likelihood of tumor immune escape (Wu et al., 2021; Zeng et al., 2022); therefore, the higher the IPS and the lower the TIDE score, the better the patient’s outcome to ICIs. This study showed that patients with gastric cancer in the low-risk group had higher IPS scores and lower TIDE scores, suggesting that patients in the low-risk group are highly immunogenic, again demonstrating that patients in the low-risk group are a potentially highly beneficial population for ICIs treatment. In addition, we analyzed the differences in sensitivity of common antitumor drugs between high- and low-risk groups to provide a new perspective on clinical antitumor drug combination strategies.

Our study also has some limitations. This study is a retrospective study based on public data and needs to be further validated in a large, multicenter prospective study. Second, this study needs to incorporate more clinicopathological features for a more comprehensive analysis of the clinical value of the risk model. In addition, *in vivo and in vitro* experiments are needed to further explore the specific mechanisms of risk scores in the TME.

## Conclusion

In this study, we found that TGF-β cluster A presented an immunosuppressive microenvironment with shorter OS. Second, we constructed a risk model associated with TSRGs to predict the prognosis of gastric cancer patients. In addition, gastric cancer patients in the low-risk group, characterized by higher TMB, the proportion of MSI-H, IPS, and lower TIDE score, may be more sensitive to immunotherapy.

## Data Availability

The original contributions presented in the study are included in the article/Supplementary Material, further inquiries can be directed to the corresponding authors.

## Supplementary material

Supplementary figure S1 Schematic diagram of the study design.

Supplementary figure S2 Risk model validation on the imvigor210 cohort. **(A)** Kaplan–Meier survival analysis between high- and low-risk groups. **(B)** Immunotherapy response analysis. CR: Complete response; PR: Partial response; SD: Stable disease; PD: Progressive disease.

## Abbreviations

CNV, copy number variation; CR, Complete response; DEGs, differentially expressed genes; EMT, epithelial-mesenchymal transition; FPKM, fragments per kilobase million; GEO, gene expression omnibus; GO, gene ontology; GSVA, gene set variation analysis; ICIs, immune checkpoint inhibitors; IC50, half inhibitory centration; IPS, immunophenoscore; KEGG, kyoto encyclopedia of genes and genomes; LASSO, least absolute shrinkage and selection operator; MSigDB, molecular signatures database; MSI-H, high microsatellite instability; OS, overall survival; PD, Progressive disease; PR, Partial response; SD, Stable disease; ssGSEA, single sample gene set enrichment analysis; TCGA, the cancer genome atlas; TIDE, tumor immune dysfunction and exclusion; TILs, tumor-infiltrating lymphocytes; TMB, tumor mutation burden; TME, tumor microenvironment; TPM, transcripts per kilobase million; TSRGs, TGF-β signaling pathway-related genes.
