# Supplementary figures and images for: Identification of TGF-β signaling-related molecular patterns, construction of a prognostic model, and prediction of immunotherapy response in gastric cancer
Source: Front Pharmacol. 2022 Nov 18;13:1069204. doi: 10.3389/fphar.2022.1069204 (PMC9715605; doi:10.3389/fphar.2022.1069204)

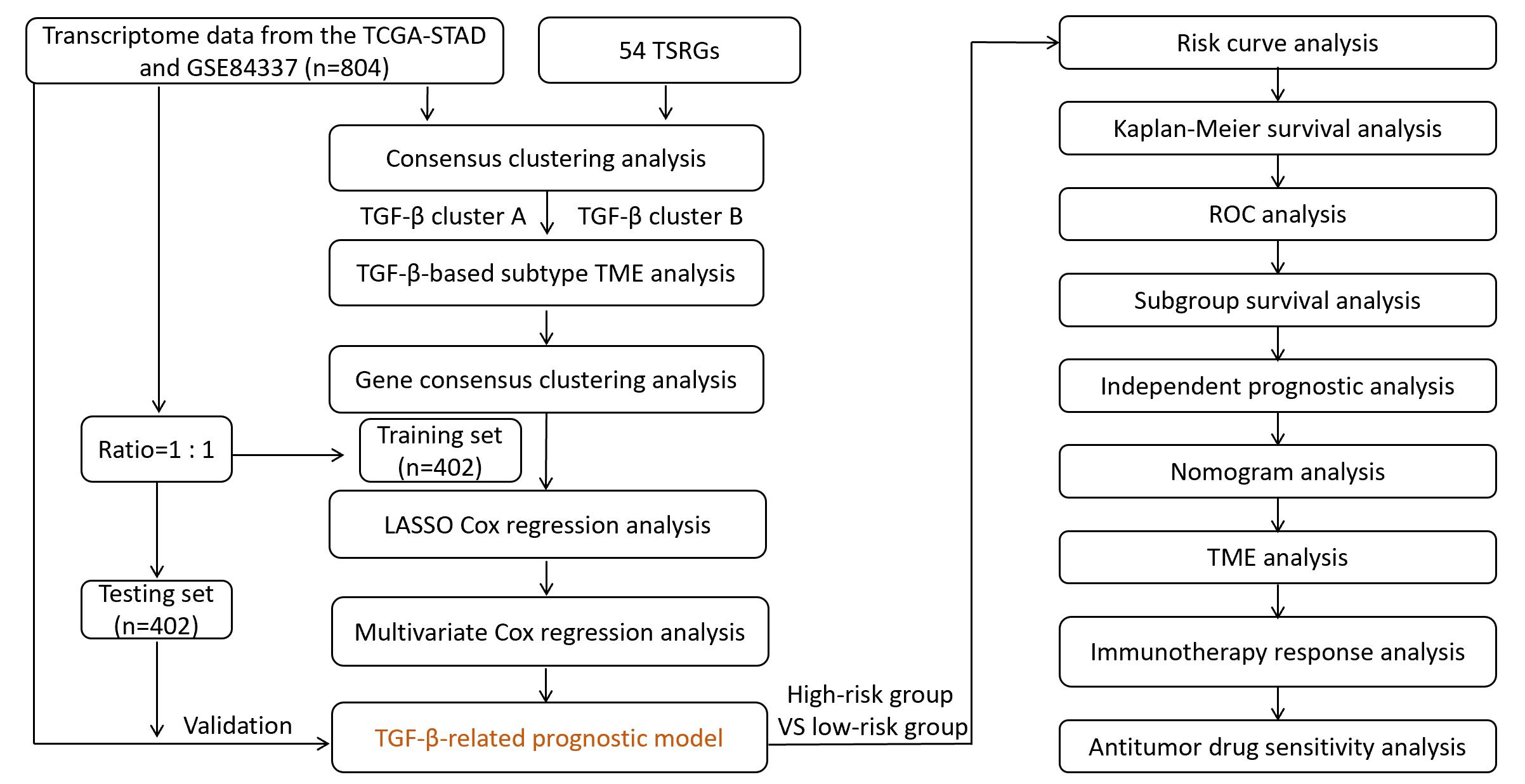

Supplement: Supplementary file 1 [file Image1.JPEG]

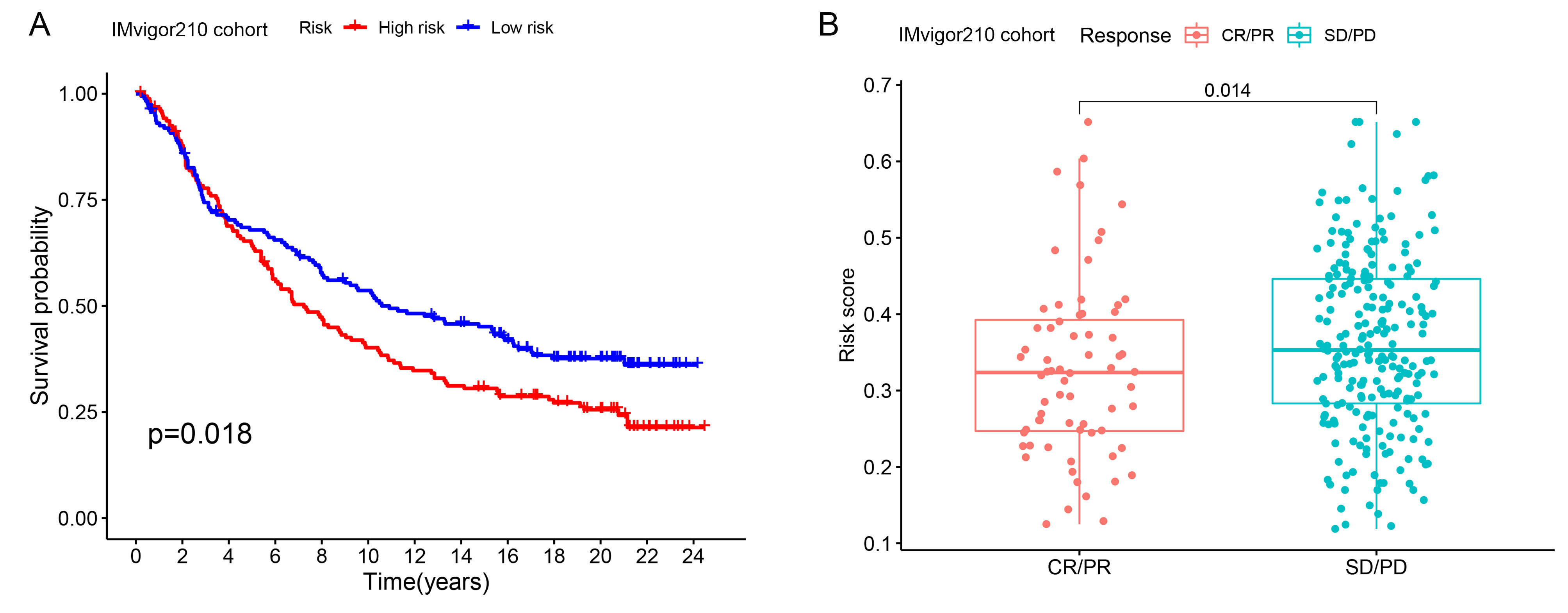

Supplement: Supplementary file 2 [file Image2.JPEG]
